# Effect of cytoglobin overexpression on extracellular matrix component synthesis in human tenon fibroblasts

**DOI:** 10.1186/s40659-019-0229-4

**Published:** 2019-04-16

**Authors:** Haiying Wei, Lili Lin, Xiaomei Zhang, Zhuolei Feng, Yeqing Wang, Yan You, Xiaodan Wang, Yongsheng Hou

**Affiliations:** 1 Department of Ophthalmology, The First Affiliated Hospital, Harbin Medical University, 23 Youzheng Street, Harbin, 150001 Heilongjiang Province People’s Republic of China; 2 Department of Dermatology, The Fourth Affiliated Hospital, Harbin Medical University, Harbin, China

**Keywords:** Glaucoma, Cytoglobin, Human tenon fibroblast, Extracellular matrix

## Abstract

**Background:**

Conjunctival filtering bleb scar formation is the main reason for the failure of glaucoma filtration surgery. Cytoglobin (Cygb) has been reported to play an important role in extracellular matrix (ECM) remodeling, fibrosis and tissue damage repairing. This study aimed to investigate the role of Cygb in anti-scarring during excessive conjunctival wound healing after glaucoma filtration surgery.

**Methods:**

Cygb was overexpressed in human tenon fibroblasts (hTFs) by transfecting hTFs with lentiviral particles encoding pLenti6.2-FLAG-Cygb. Changes in the mRNA and protein levels of fibronectin, collagen I, collagen III, TGF-β1, and HIF1α were determined by RT-PCR and western blotting respectively.

**Results:**

After Cygb overexpression, hTFs displayed no significant changes in visual appearance and cell counts compared to controls. Whereas, Cygb overexpression significantly decreased the mRNA and protein expression levels of collagen I, collagen III and fibronectin compared with control (p < 0.01). There was also a statistically significant decrease in the mRNA and protein levels of TGF-β1 and HIF-1α in hTFs with overexpressed Cygb compared with control group (p < 0.05).

**Conclusion:**

Our study provided evidence that overexpression of Cygb decreased the expression levels of fibronectin, collagen I, collagen III, TGF-β1 and HIF-1α in hTFs. Therefore, therapies targeting Cygb expression in hTFs may pave a new way for clinicians to solve the problem of post-glaucoma surgery scarring.

**Electronic supplementary material:**

The online version of this article (10.1186/s40659-019-0229-4) contains supplementary material, which is available to authorized users.

## Introduction

Glaucoma is the second leading cause of blindness worldwide, besides, the blindness caused by glaucoma is irreversible [1]. It is reported that glaucoma presently affects 60.5 million people, and the prevalence is projected to increase to 79.6 million by 2020 worldwide. Glaucoma filtering surgery (GFS) is considered as an effective treatment for glaucoma, but postoperative filtering bleb scar formation often leads to surgical failure. Mytomycin-C (MMC) and 5-fluorouracil (5-FU) have been used intraoperatively and postoperatively in patients undergoing GFS, but the serious complications, such as filtering bleb leaks and low-pressure cystoid macular edema, limits their application [2]. Therefore, prevention of scarring post glaucoma surgery has become an important subject of study in ophthalmology. A previous study showed that human tenon fibroblasts (hTFs), an undifferentiated mesenchymal cell type found in the connective tissue of the conjunctiva, play an important role in the process of scarring post GFS [3]. Therefore, hTFs may have important significance in inhibiting filtering bleb scar formation post GFS.

Cytoglobin (Cygb), also known as stellate cell activation-associated protein, was originally characterized as a heme protein that exhibits enhanced expression in stellate cells in a rat liver fibrosis model [4]. Cygb is a new member of the oxygen-carrying globulin family and is widely expressed in organisms. In the human genome, Cygb gene is located on chromosome 17q25.3, containing three introns and four exons, and encoding a protein composed of 190 amino acids [5, 6]. It has been demonstrated that Cygb is expressed in a broad range of tissues, including the brain, heart, liver, and lung, and in different developmental stages. Additionally, Cygb is expressed more in connective tissue than in other tissues, and is abundant in fibroblasts and fibroblast cell lines, such as cartilage cells, osteoblasts, hepatic stellate cells, and myofibroblasts. Recently, some studies have demonstrated that Cygb is also expressed at a low level in neurons, muscle cells, liver parenchymal cells, and epithelial cells [7, 8] as well as in iris, ciliary body, retina and cornea of the human eye [9, 10]. Thuy et al. [11] suggested that Cygb-deficient mice display multiple organ abnormalities, such as cardiac enlargement, liver fibrosis, and lymphoma. Xu et al. [12] reported that normal overexpression of Cygb during tissue injury played a homeostatic effect, which could inhibit free radical-induced fibroblast activation and tissue fibrosis. Postoperative filtering bleb scars result from fibroblast proliferation and subconjunctival fibrosis characterized by cell proliferation, migration, and differentiation, excessive ECM synthesis and deposition, free radical accumulation, as well as local inflammation characterized by the presence of various cytokines, such as TGF, vascular endothelial growth factor (VEGF), and basic fibroblast growth factor (bFGF) [15, 16]. Previous studies have shown that activation, proliferation, transformation, and secretion of cytokines, as well as synthesis of ECM components, like collagen I and collagen III, in hTFs are the core links in the process of postoperative scarring [16–19]. As a new member of the oxygen-carrying globulin family, Cygb is specifically expressed in fibroblasts and the derivative cells, implying that Cygb is closely related to organ fibrosis. Considerable studies have revealed that Cygb participates in fibrosis formation through its antioxidant function. Specially, Cygb is involved in ECM remodeling and the Cygb expression parallels the expression of ECM proteins. Thus, we speculated that Cygb may play a role in anti-scarring therapy during excessive conjunctival wound healing after GFS.

Therefore, in this study, we investigated the changes in the components of the extracellular matrix (ECM) after Cygb overexpression and the effect of Cygb overexpression on HIF-1α and TGF-β1 expression. Our findings may provide clinicians with new therapeutic targets to better treat post-glaucoma surgery scarring.

## Materials and methods

### Cell culture

Small tenon biopsy specimens were obtained from patients who underwent standard intraocular surgery as described previously [13]. All patients had provided written consent before conduction of the study. This study was approved by the institutional review board/ethics committee. Primary hTFs were obtained in an expansion culture of the human tenon explants and cultured in Dulbecco’s modified Eagle’s medium (DMEM; Biochrom, Berlin, Germany) at 37 °C with 5% CO_2_. The medium was supplemented with 0.2% fetal calf serum, 3.125 mL/L l-glutamine, and 2.5 mL/L penicillin/streptomycin (Biochrom, Berlin, Germany). The culture medium was changed every other day unless otherwise stated.

### Plasmid construction and lentiviral infection

To generate Cygb expression vectors, Cygb was amplified from HEK293T cDNA by PCR and cloned into a pLenti6.2-FLAG lentiviral vector. pLenti6.2-FLAG hTFs were used as negative control.

The hTFs were transfected with lentiviral particles encoding pLenti6.2-FLAG-Cygb. To generate lentiviral particles, the HEK293T cells were co-transfected with an envelope plasmid (pLP/VSVG), a packaging vector (psPAX2), and a cDNA expression vector (Cygb) using Lipofectamine 2000. At 24 to 48 h post transfection, the medium containing the lentiviral particles was harvested, filtered, and used to infect the hTFs. After 24 h post-transduction, the hTFs were stably selected in the presence of 5 µg/mL blasticidin.

### Cell morphology evaluation

The cellular morphological changes of hTFs after cell transfection were observed using phase-contrast microscopy (Nikon, Japan). Briefly, hTFs (1 × 10^5^) transfected with pLenti6.2-FLAG-Cygb or pLenti6.2-FLAG were plated into 6-well culture plates. These cells were then incubated in DMEM in 5% CO_2_ at 37 °C, reaching sub-confluence in the plates. After 24 h, HTFs were monitored using an inverted phase-contrast light microscope equipped with a photographic system.

### Real-time polymerase chain reaction (RT-PCR) analysis

Total RNA was isolated using TRIzol reagent (Invitrogen, Carlsbad, CA, USA) according to the manufacturer’s instructions. Complementary DNA was synthesized using AMV reverse transcriptase at 42 °C for 1 h. The mixture was then boiled for 5 min to inactivate the reverse transcriptase and then chilled quickly on ice. The synthesized cDNAs were analyzed by real-time PCR using a QuantiTect SYBR Green PCR Kit on a Rotor-Gene 6000 (Corbett Life Science, Sydney, NSW, Australia). The primers used in this study are listed in Additional file 1: Table S1.

### Western blotting

Protein samples (10 mg) were subjected to sodium dodecyl sulfate polyacrylamide gel electrophoresis (SDS-PAGE) and electrically transferred onto Immobilon P membranes (Millipore Corp., Bedford, MA, USA). The membranes were blocked with 5% skim milkand incubated with primary antibodies including lamin B (1:1000; 66095-1-Ig; ProteinTech Group, Chicago, IL, USA), GAPDH (1:1000; M20005 M; Abmart, China), FLAG (1:1000; F1804; Sigma, St. Louis, MO, USA), hypoxia-inducible factor-1 alpha (HIF-1α) (1:1000; ab51608; Abcam, Cambridge, MA, USA), transforming growth factor (TGF)-β1 (1:1000; ab179695; Abcam), fibronectin (1:1000; ab32419; Abcam), collagen III (1:1000; BA0326; Boster, Wuhan, China), and collagen I (1:1000; BA0325; Boster). Then membranes were incubated with peroxidase-conjugated secondary antibodies. Immunoreactive bands were visualized using an enhanced chemiluminescence system (Amersham, Buckingshamshire, UK). The density of each band was analyzed by a GS-700 Imaging Densitometer (BIO-RAD, Hercules, CA, USA).

### Statistical analysis

All experiments were repeated three times. The results of multiple experiments are expressed as mean ± standard deviation (SD). Statistical analyses were performed using SPSS 19.0 (SPSS Inc., Chicago, IL, USA). p value was calculated using student’s t test. Statistical significance was set at p < 0.05.

## Results

### Effect of Cygb overexpression on morphology of hTFs

**Fig. 1:**
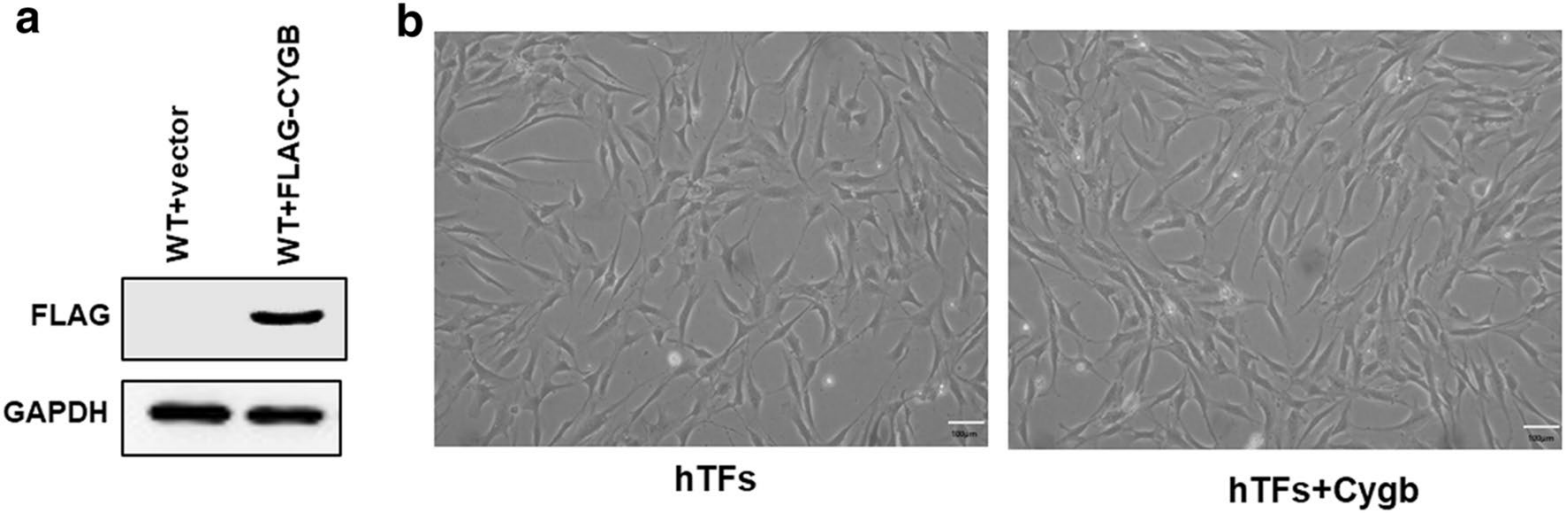
Morphological examination of wild type (WT) and FLAG-Cygb human tenon fibroblasts (hTFs). **a** Western blot analysis of FLAG and GAPDH (as control) in WT and FLAG-Cygb hTFs. **b** Cells were imaged at 24 h post-seeding. The scale bar represents 100 μm

To investigate the function of Cygb in hTFs, we transfected hTFs with lentiviral particles encoding pLenti6.2-FLAG-Cygb or pLenti6.2-FLAG as control (Fig. 1a). The cellular morphology observation of hTFs after Cygb overexpression under phase-contrast microscopy revealed that there was no significant change in visual appearance of hTFs compared with control group. Additionally, there was also no significant changes in cell counts after Cygb overexpression (Fig. 1b and Additional file 2: Fig. S1).

### Effect of Cygb overexpression on ECM component in hTFs

**Fig. 2:**
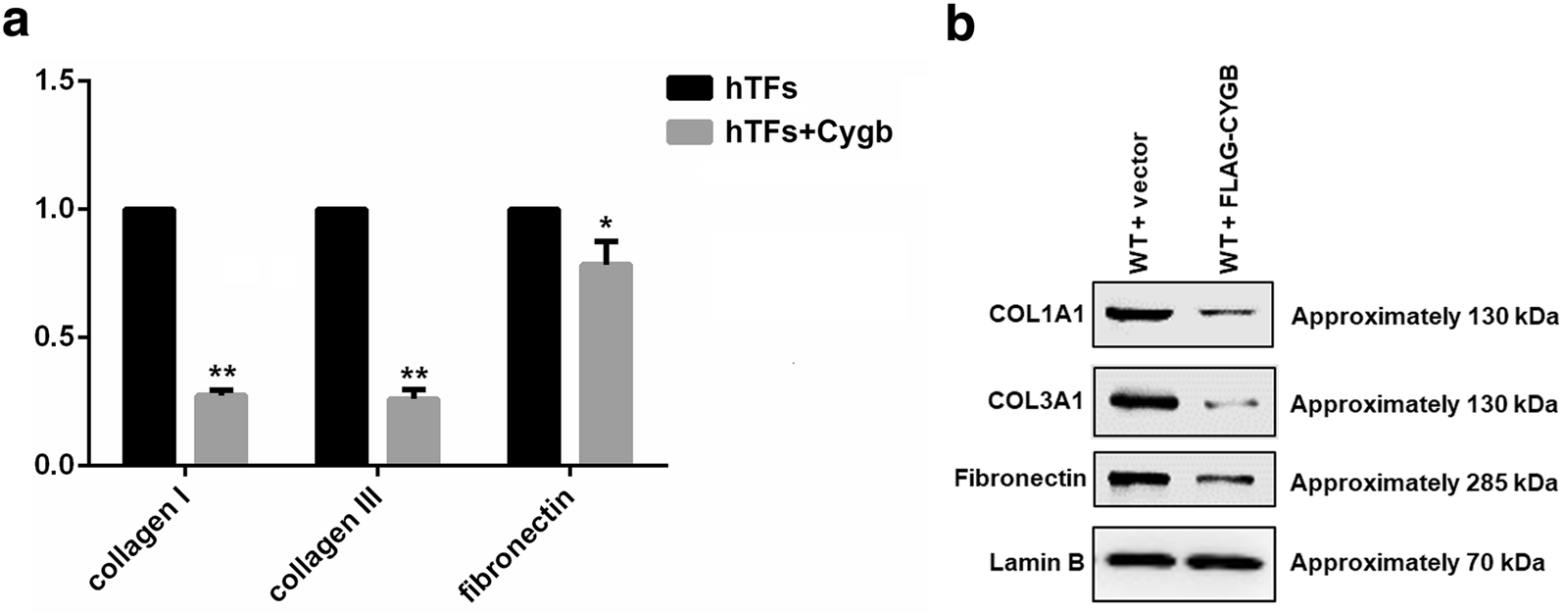
**a** RT-PCR analysis of mRNA expression levels of collagen I, collagen III and fibronectin in human tenon fibroblasts after Cygb overexpression. Data are represented as the mean ± standard deviation (n = 3), *p < 0.05; **p < 0.01, t test. **b** Western blot analysis of collagen I, collagen III, fibronectin and lamin B (as control) in human tenon fibroblasts after Cygb overexpression

To determine the effect of Cygb on the synthesis of ECM components in hTFs, we assessed the transcriptional activities of collagen I, collagen III and fibronectin after Cygb overexpression. As shown in Fig. 2a, there was a significant decrease in the mRNA expression levels of collagen I, collagen III (p < 0.01) and fibronectin (p < 0.05) compared to control cells. Similarly, the protein levels of collagen I, collagen III and fibronectin were also decreased in Cygb overexpression group compared with that in control group (Fig. 2b).

### Effect of Cygb overexpression on cytokines in hTFs

**Fig. 3:**
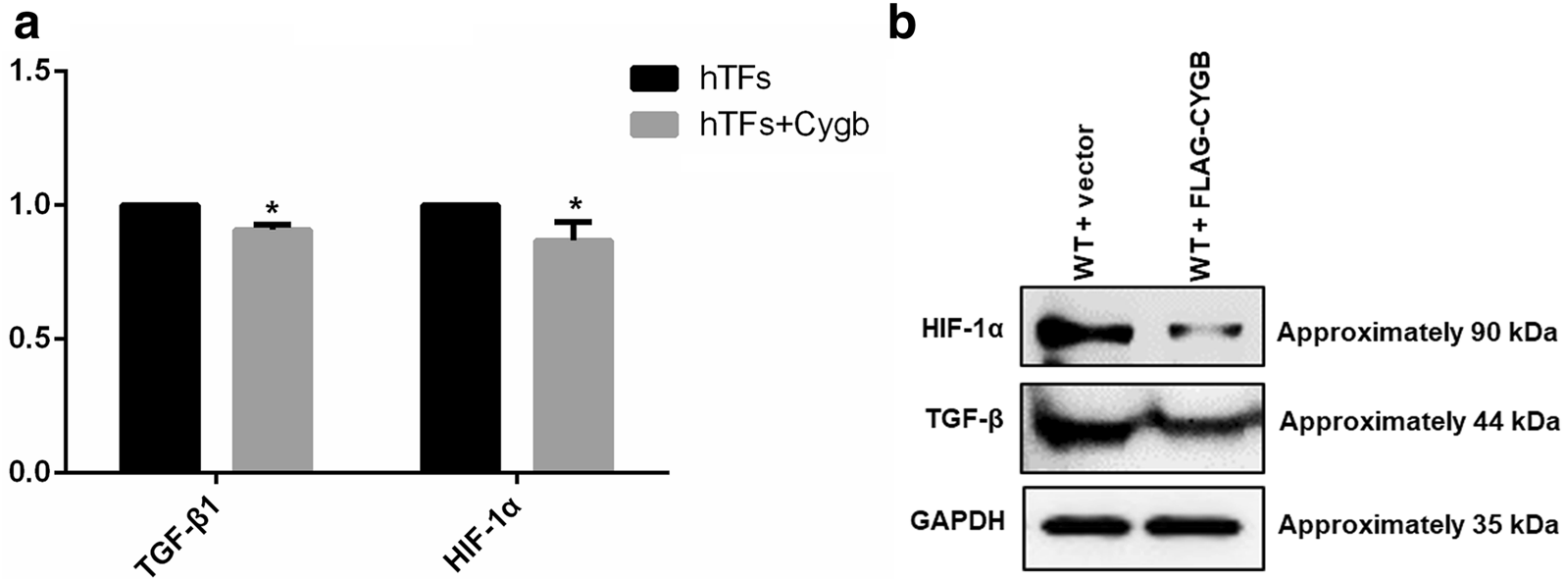
**a** RT-PCR analysis of mRNA expression levels of TGF-β and HIF-1α in human tenon fibroblasts after Cygb overexpression. Data are represented as the mean ± standard deviation (n = 3), *p < 0.05, t test. **b** Western blot analysis of TGF-β, HIF-1α and GAPDH (as control) in human tenon fibroblasts after Cygb overexpression

To investigate the cytokines involved in regulating fibronectin, collagen I and collagen III production in Cygb-overexpressing hTFs, we quantified the mRNA expression levels of TGF-β1 and HIF-1α by RT-PCR. As presented in Fig. 3a, the mRNA levels of TGF-β1 and HIF-1α were significantly decreased in Cygb-overexpressing hTFs compared to control cells (p < 0.05). Moreover, the decrease of TGF-β1 and HIF-1α expression in Cygb overexpression group was also confirmed at the protein level detected by western blotting (Fig. 3b).

## Discussion

GFS is one of the main therapeutic methods for glaucoma clinically. Nevertheless, postoperative scarring has been considered as the main reason for the failure of GFS [14]. Postoperative filtering bleb scars result from fibroblast proliferation and subconjunctival fibrosis characterized by cell proliferation, migration, and differentiation, excessive ECM synthesis and deposition, free radical accumulation, as well as local inflammation characterized by the presence of various cytokines, such as TGF, vascular endothelial growth factor (VEGF), and basic fibroblast growth factor (bFGF) [15, 16]. Previous studies have shown that activation, proliferation, transformation, and secretion of cytokines, as well as synthesis of ECM components, like collagen I and collagen III, in hTFs are the core links in the process of postoperative scarring [16–19].

As a new member of the oxygen-carrying globulin family, Cygb is specifically expressed in fibroblasts and the derivative cells, implying that Cygb is closely related to organ fibrosis. Considerable studies have revealed that Cygb participates in fibrosis formation through its antioxidant function [4, 8, 20, 21]. Specially, Cygb is involved in ECM remodeling and the Cygb expression parallels the expression of ECM proteins. In turn, ECM components, such as laminin and collagen I, can also regulate the expression of Cygb through the integrin signaling pathway. Signaling mediated by ECM provides a potential feedback mechanism to regulate cell function, which suggested that Cygb may be a downstream target of the integrin signaling pathway [22, 23]. It has been reported that in the process of liver fibrosis, the protein and mRNA expression levels of Cygb increase progressively [24]. Normal overexpression of Cygb during tissue injury has a homeostatic effect, inhibiting free radical-induced fibroblast activation and tissue fibrosis [12]. In the present study, after Cygb was overexpressed in hTFs, significant decrease was observed in ECM component synthesis, which was in accordance with the studies above. Therefore, Cygb may represent a novel target in anti-fibrotic treatments.

Recently, Cygb expression is reported to be influenced by hypoxia, and hypoxia could increase the protein expression level of Cygb in several tumor cell lines [25]. Under hypoxic conditions, HIF-1α could combine with the hypoxia response element that localizes at the 5′UTR of the Cygb gene, and directly promote Cygb transcription [26]. During postoperative wound healing, vasodilatation and angiogenesis are required to meet blood supply requirement [27], thereby resulting in the production of many vasoactive factors and angiogenesis factors, such as VEGF. Interestingly, production of VEGF is implemented through HIF-1 [28]. HIF-1 consists of two subunits, HIF-1α and HIF-1β. HIF-1β is relatively stable under normoxic conditions, while HIF-1α rapidly degrades under normoxic conditions. Conversely, hypoxia increases HIF-1α activity and induces HIF-1α protein accumulation. A recent study reported that in indomethacin-induced injury, HIF-1α protein level was significantly increased in the early healing phase, while Cygb protein level was significantly increased in the late phase. These findings suggest that HIF-1α and Cygb are expressed at different times but before angiogenesis, further indicating that VEGF is expressed after Cygb and HIF-1α expression [29].

In addition, Cygb has antioxidant functions, which is able to sense oxygen concentrations and acts as a regulatory protein to protect cells from reactive oxygen species [30]. Trent and Hargrove [31] reported that Cygb could increase the adaptive response of the renal mitochondrial respiratory chain to adapt hypoxic conditions in kidney. Nishi et al. [32] recently established transgenic rats constitutively overexpressing Cygb and found that Cygb could resist oxidative stress but does not promote cell proliferation. They believed that the loss and distortion of capillaries led to decreased oxygen diffusion efficiency and the upregulated expression of Cygb is due to hypoxia-induced decrease in oxygen tension. Additionally, Nishi et al. [32] have also proved that Cygb involves in the fibrotic process and inhibits the synthesis of collagen in immortalized kidney fibroblasts, which is consistent with our finding that upregulation of Cygb in hTFs suppresses the synthesis of collagen I and III.

## Conclusion

In conclusion, our results identified no obvious changes in cell morphology and cell counts, but decreased expression of collagen I, collagen III and fibronectin as well as HIF-1α and TGF-β1 after Cygb overexpression in hTFs, which indicated that targeting Cygb in hTFs may influence the synthesis of ECM components. However, change in the expression level of Cygb after GFS is still unclear and remains to be confirmed in further study. Taken together, Cygb may involve in the process of GFS scarring and further studies on its action mechanism and therapeutic potential may help us to determine its clinical significance.

## Additional files

**Additional file 1: Table S1.** List of RT-PCR primers used in this study.

**Additional file 2: Fig. S1.** Immunostaining in Cygb overexpression group and control group. Intracellular protein signal was detectable in Cygb overexpression group but not in control group.

## Abbreviations

Cygb: cytoglobin
ECM: extracellular matrix
hTFs: human tenon fibroblasts
GFS: glaucoma filtering surgery
MMC: mytomycin-C
5-FU: 5-fluorouracil
bFGF: basic fibroblast growth factor
SD: standard deviation
VEGF: vascular endothelial growth factor
