# Supplementary material for: Effect of cytoglobin overexpression on extracellular matrix component synthesis in human tenon fibroblasts
Source: Biol Res. 2019 Apr 16;52:23. doi: 10.1186/s40659-019-0229-4 (PMC6466771; doi:10.1186/s40659-019-0229-4)
Supplement: Supplementary file 1 — Additional file 1: Table S1. List of RT-PCR primers used in this study. [file 40659_2019_229_MOESM1_ESM.docx]

**Additional data**

Table S1. List of RT-PCR primers used in this study.

| **Primer Names** | **Sequences (5’- to -3’)** |
| --- | --- |
| Collagen I-F | CCAGGCAGAGATGGTGAAGA |
| Collagen I-R | GCAGGTCCTTGGAAACCTTG |
| Collagen III-F | GAGAACCTGGTGCAAATGGG |
| Collagen III-R | TTCCCATCACTTCCTGGTCC |
| Fibronectin-F | GTGCCACTTCCCCTTCCTAT |
| Fibronectin-R | ATCCCACTGATCTCCAATGC |
| TGF-β1-F | TACAGCAACAATTCCTGGCG |
| TGF-β1-R | GCAGTGTGTTATCCCTGCTG |
| HIF-1α-F | TCCAAGAAGCCCTAACGTGT |
| HIF-1α-R | TGATCGTCTGGCTGCTGTAA |
| GAPDH-F | AACGGATTTGGTCGTATTG |
| GAPDH-R | GGAAGATGGTGATGGGATT |
